# Gallbladder papillary neoplasms share pathological features with intraductal papillary neoplasm of the bile duct

**DOI:** 10.18632/oncotarget.16360

**Published:** 2017-03-18

**Authors:** Xueshuai Wan, Jie Shi, Anqiang Wang, Yuan Xie, Xiaobo Yang, Chengpei Zhu, Haohai Zhang, Liangcai Wu, Shanshan Wang, Hanchun Huang, Jianzhen Lin, Yongchang Zheng, Zhiyong Liang, Xinting Sang, Haitao Zhao

**Affiliations:** ^1^ Department of Liver Surgery, Peking Union Medical College Hospital, Chinese Academy of Medical Sciences and Peking Union Medical College, Beijing, China; ^2^ Department of Pathology, Peking Union Medical College Hospital, Chinese Academy of Medical Sciences and Peking Union Medical College, Beijing, China

**Keywords:** gallbladder, papillary, mucin, cytokeratin, CDX2

## Abstract

Intraductal papillary neoplasm of the bile duct (IPNB) has been widely recognized. However, the knowledge of intracystic papillary neoplasm of the gallbladder (IPNG) including papillary adenoma and adenocarcinoma is not well defined. In this study, we compared the clinicopathological and immunohistochemical features between 32 IPNG cases and 32 IPNB cases. IPNG-1 (low-high grade dysplasia) exhibited an earlier onset age, smaller tumor size and lower level of CK20 expression compared to IPNG-2 (invasive carcinoma). Histologically, pancreaticobiliary and intestinal subtype accounted for nearly half of IPNG or IPNB (44.4% and 48.1% vs. 44.0% and 44.0%), respectively. Immunohistochemically, 88.9% of IPNG and 92.0% of IPNB cases were positive for MUC1, and 96.3% and 92.0% for CK7, respectively. CDX2 and MUC2 were more highly expressed in the intestinal subtype than in other subtypes. CK20 expression increased in parallel with tumor progression. In addition, 53.1% of IPNG cases and 68.6% of IPNB cases exhibited invasive carcinoma, and showed significant survival advantages to conventional gallbladder adenocarcinoma and cholangiocarcinoma, respectively. In conclusion, papillary adenoma and adenocarcinoma of the gallbladder can be recognized as different pathological stages of IPNG, and they share pathological features with IPNB.

## INTRODUCTION

Intraductal papillary neoplasm of the bile duct (IPNB) is a class of tumor that is characterized by predominant intraductal papillary growth that may occur anywhere along the biliary tree [1–8]. Its’ definition does not include tumors originating from the gallbladder. However, some gallbladder neoplasms exhibit a papillary growth pattern that projects into the lumen of the gallbladder, including papillary adenocarcinoma and adenoma. Adsay et al. examined mass-forming (≥ 1 cm) pre-invasive neoplasms of the gallbladder and found that these tumors exhibited a spectrum of dysplastic changes, variable configuration, and different levels of MUCs, CKs, and CDX2 expression in different cell lineages, which resembled the presentation of IPNB [9]. However, the authors included tubular neoplasms of the gallbladder in their research, and they did not conduct comparative analyses with IPNB.

Therefore, we selected gallbladder papillary adenocarcinoma, adenoma and IPNB cases from the same population and compared their clinicopathological features, histological subtypes and expression profile of MUCs, CKs, and CDX2 to investigate whether papillary adenoma and papillary adenocarcinoma of the gallbladder can be recognized as different pathological stages of the same disease, which we named intracystic papillary neoplasm of the gallbladder (IPNG), and whether papillary tumors from the gallbladder and bile duct exhibited similar pathological features.

## RESULTS

**Figure 1:**
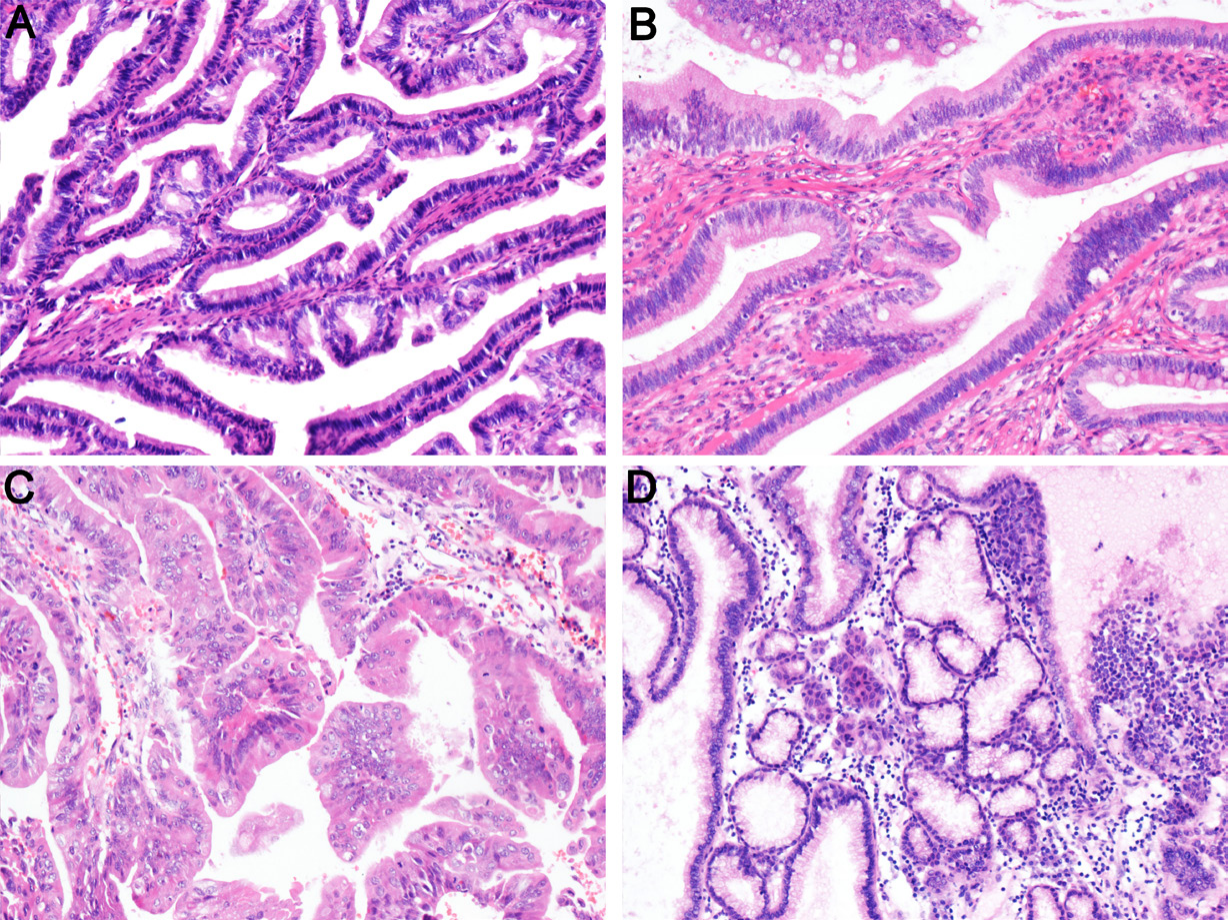
Histological subtypes identified in IPNG and IPNB (**A**) pancreaticobiliary; (**B**) intestinal; (**C**) oncocytic; (**D**) gastric. H&E staining 100×.

**Figure 2:**
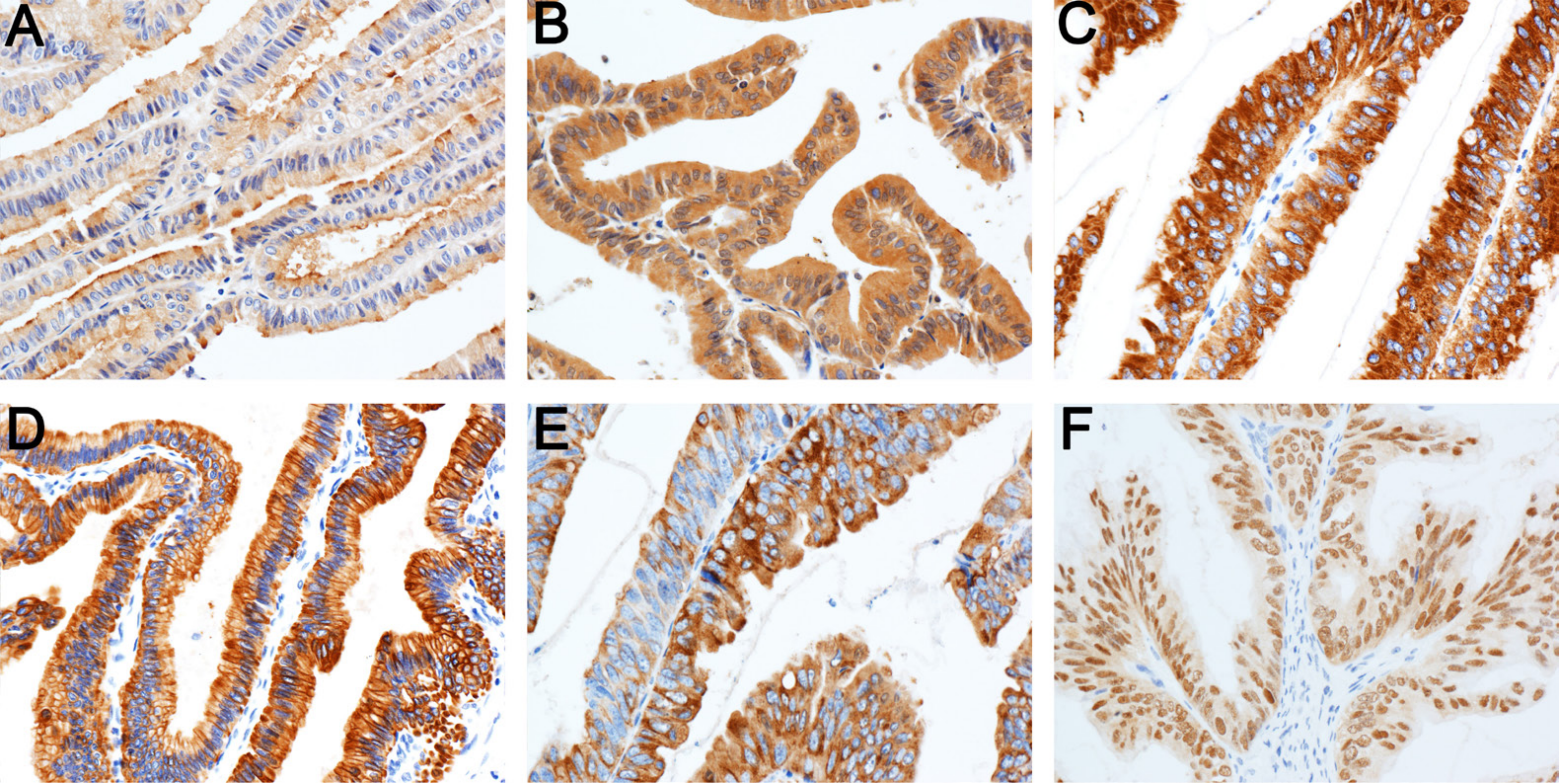
Expression of immunohistochemical markers in tumor cells MUC1 in the apical membrane (**A**) and cytoplasm of tumor cells (**B**) MUC2 (**C**) CK7 (**D**) and CK20 (**E**) in the cytoplasm; CDX2 in the nucleus (**F**). Immunohistochemical staining 200×.

Thirteen of the 27 cases of IPNG were identified as intestinal, 12 cases as pancreaticobiliary, and 2 cases as oncocytic subtype. The numbers of intestinal, pancreaticobiliary, and oncocytic subtypes in the 25 cases of IPNB were 11, 11, and 3, respectively (Table 1). None of the IPNG or IPNB cases in the present cohort were classified as gastric subtype. However, three cases of IPNG exhibited a minor component of gastric cells (Figure 1). A total of 59.3% of IPNG (16 cases) and 48% (12 cases) of IPNB contained two or more histological subtypes of tumor cells. MUC1 was expressed primarily in the apical membrane and occasionally in the cytoplasm of tumor cells. MUC2, CK7, and CK20 were present primarily in the cytoplasm, and CDX2 was located in the nucleus (Figure 2).

**Table 1:** Comparison of clinicopathological data among groups of IPNG and IPNB

	IPNG			IPNB			*P* value
	IPNG-1	IPNG-2	*P* value	IPNB-1	IPNB-2	*P* value	
Demographic							
Age (year)	56.5 ± 16.9	65.8 ± 17.2	*0.135*	59.7 ± 1 1.5	56.1 ± 12.7	*0.445*	0.261
Gender (M:F)	8:7	6:11	*0.476*	6:4	11:11	*0.712*	0.453
Symptoms							
Abdominal pain	8/15§	8/17	*1.000*	5/10§	7/22	*0.438*	0.313
Jaundice	1/15	2/17	*1.000*	4/10	16/22	*0.119*	< 0.001
Fever	2/15	3/17	*1.000*	4/10	5/22	*0.407*	0.226
Weight loss	3/15	6/17	*0.444*	5/10	12/22	*1.000*	0.042
Biliary stones	7/15	6/17	*0.720*	3/10	1/22	*0.079*	0.011
Liver function tests							
ALT	1/15	2/16	*1.000*	5/10	17/22	*0.217*	< 0.001
TBil	1/15	3/16	*0.600*	6/10	14/22	*1.000*	< 0.001
DBil	1/15	2/16	*1.000*	5/10	15/22	*0.438*	< 0.001
GGT	4/15	3/16	*0.685*	6/10	20/22	*0.060*	< 0.001
ALP	1/15	2/16	*1.000*	6/10	15/22	*0.703*	< 0.001
Tumor markers							
CEA	0/7	4/13	*0.249*	1/10	4/22	*1.000*	0.977
CA19-9	2/7	2/14	*0.574*	5/10	15/21	*0.423*	0.001
Pathological features							
Multiple lesions (≥ 2)	4/15	5/17	*1.000*	2/10	4/22	*1.000*	0.376
Size (cm)	1.5 ± 1.2	4.0 ± 2.2	*< 0.001*	2.3 ± 1.3	2.3 ± 1.1	*0.944*	0.231
Lymphatic metastasis	1/14	4/17	*0.344*	0/10	4/21	*0.227*	1.000
Positive margin	0/15	3/17	*0.229*	0/10	5/22	*0.155*	0.705
Histological subtype			*1.000*			*0.019*	0.923
Pancreaticobiliary	5/12	7/15		7/9	4/16		
Intestinal	6/12	7/15		1/9	10/16		
Oncocytic	1/12	1/15		1/9	2/16		
Gastric	0/12	0/15		0/9	0/16		

Note. §Number of positive cases /total number of cases with available data.

Abbreviations: ALT, alanine aminotransferase; TBil, total bilirubin; DBil, direct bilirubin; GGT, γ-glutamyl transpeptidase; ALP, alkaline phosphatase; CEA, carcinoembryonic antigen; CA19-9, carbohydrate antigen 19-9.

### Comparison of IPNB-1 and IPNB-2

### Clinicopathological features

IPNB-1 and IPNB-2 exhibited nearly identical clinicopathological features (Table 1).

### Histological subtype and immunohistochemical staining

**Figure 3:**
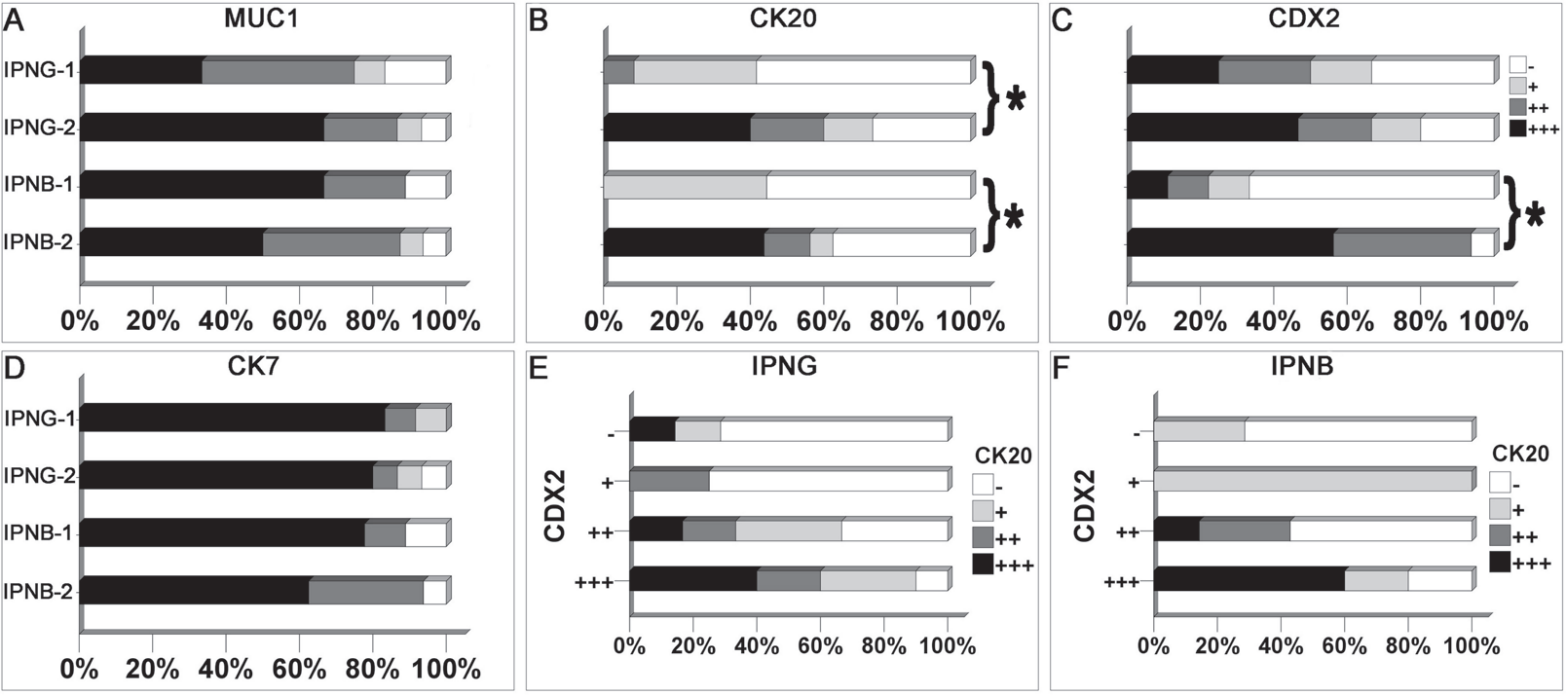
Expression profile of immunohistochemical markers in IPNG and IPNB MUC1 (**A**) and CK7 (**D**) were diffusely expressed in most cases of IPNG and IPNB. CK20 (**B**) was more frequently expressed in IPNG-2 and IPNB-2 than IPNG-1 and IPNB-1, respectively. IPNB-2 expressed more CDX2 (**C**) than IPNB-1. Meanwhile, the expression of CDX2 was positively correlated with CK20 expression in IPNG (**E**) and IPNB (**F**). * means *P* < 0.05.

Seven cases of IPNB-1 were classified as pancreaticobiliary subtype, and the major subtype of IPNB-2 was intestinal (10/16, *P* = 0.019, Table 1). MUC1 and CK7 were diffusely expressed in most IPNB-1 and IPNB-2 cases (Figure 3A, 3D). CK20 was focally expressed in 4 of 9 IPNB-1 and negative in the other 5 cases, which is significantly different from that of IPNB-2 (*P* = 0.015, Figure 3B). The distribution of CDX2 expression in IPNB-1 was 1 diffuse, 1 moderate, 1 focal, and 6 negative, and there were 9 diffuse, 6 moderate, and 1 negative cases in IPNB-2 (*P* = 0.002, Figure 3C).

### Comparison of IPNG-1 and IPNG-2

### Clinicopathological features

IPNG-1 and IPNG-2 were also similar in clinical manifestations. However, the mean age of patients with IPNG-1 was 56.5 ± 16.9 years, which was younger than that of patients with IPNG-2 (65.8 ± 17.2). Moreover, the mean tumor size of IPNG-2 was significantly larger than that of IPNG-1 (4.0 ± 2.2 vs. 1.5 ± 1.2 cm, *P* < 0.001) (Table 1).

### Histological subtype and immunohistochemical staining

Pancreaticobiliary and intestinal subtype accounted for nearly half of IPNG-1 and IPNG-2, respectively (Table 1). Ten of 12 cases of IPNG-1 and 13 of 15 cases of IPNG-2 were positive for MUC1 (*P* = 0.404, Figure 3A). All cases of IPNG-1 were positive for CK7 with diffuse expression in 10 of them. Likewise, there were 12 cases of IPNG-2 expressed CK7 diffusely (*P* = 1.000, Figure 3D). Eleven of the 15 cases of IPNG-2 expressed CK20, and 6 of these cases exhibited diffuse expression. However, 7 of 12 cases of IPNG-1 were negative for CK20 (*P* = 0.041, Figure 3B). CDX2 expression was not significantly different between the two groups (*P* = 0.724, Figure 3C).

### Comparison of IPNG and IPNB

### Clinicopathological features

IPNG and IPNB showed significantly different clinical manifestations. However, they shared similarities in pathological features. A total of 28.1% of IPNG and 18.8% of IPNB lesions were multiple (*P* = 0.376). The proportion of cases with invasive carcinoma was similar in IPNG and IPNB (53.1% vs. 68.8%, *P* = 0.200). Only 5 cases of IPNG and 4 cases of IPNB exhibited lymph node involvement (*P* = 1.000) (Table 1).

### Histological subtype and immunohistochemical staining

**Figure 4:**
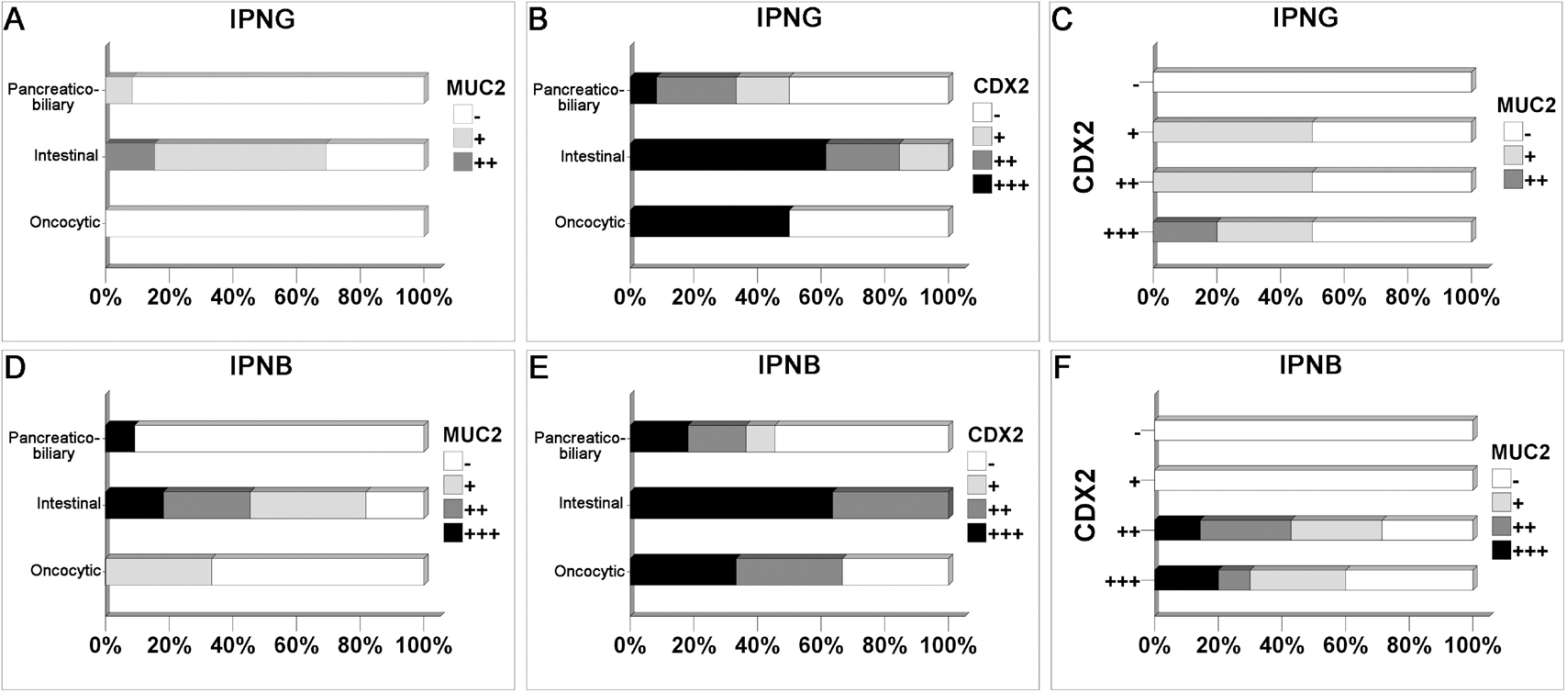
The correlation among different histological subtypes and immunohistochemical markers in IPNG and IPNB Cases with intestinal subtype expressed more MUC2 and CDX2 in IPNG (**A**, **B**) and IPNB (**D**, **E**). The expression of CDX2 was positively correlated with MUC2 expression in IPNG (**C**) and IPNB (**F**).

Most cases of pancreaticobiliary subtype in IPNG were negative for MUC2 (11/12), and 9 of 13 cases of intestinal subtype were positive (*P* = 0.005, Figure 4A). CDX2 was not expressed in half of the pancreaticobiliary subtype (6/12), and it was diffusely expressed in 8 of 13 cases of the intestinal subtype (*P* = 0.007, Figure 4B). Among the immunohistochemical markers, the expression of CDX2 was positively correlated with MUC2 and CK20, with coefficients of 0.391 (*P* = 0.044, Figure 4C) and 0.514 (*P* = 0.006, Figure 3E), respectively.

In IPNB, more cases were classified as invasive carcinoma in cases of intestinal than pancreaticobiliary subtype (90.9% vs. 36.4%, *P* = 0.033, Table 1). A total of 81.8% of intestinal cases were positive for MUC2, which contrasted with pancreaticobiliary cases, which were mostly negative for MUC2 (10/11, *P* = 0.008, Figure 4D). All intestinal cases of IPNB were positive for CDX2, 4 moderately and 7 diffusely, and 6 of the 11 pancreaticobiliary cases were negative (*P* = 0.015, Figure 4E). CDX2 expression was positively correlated with MUC2 and CK20, with coefficients of 0.451 (*P* = 0.024, Figure 4F) and 0.534 (*P* = 0.006, Figure 3F), respectively.

### Outcome and prognostic factors

**Figure 5:**
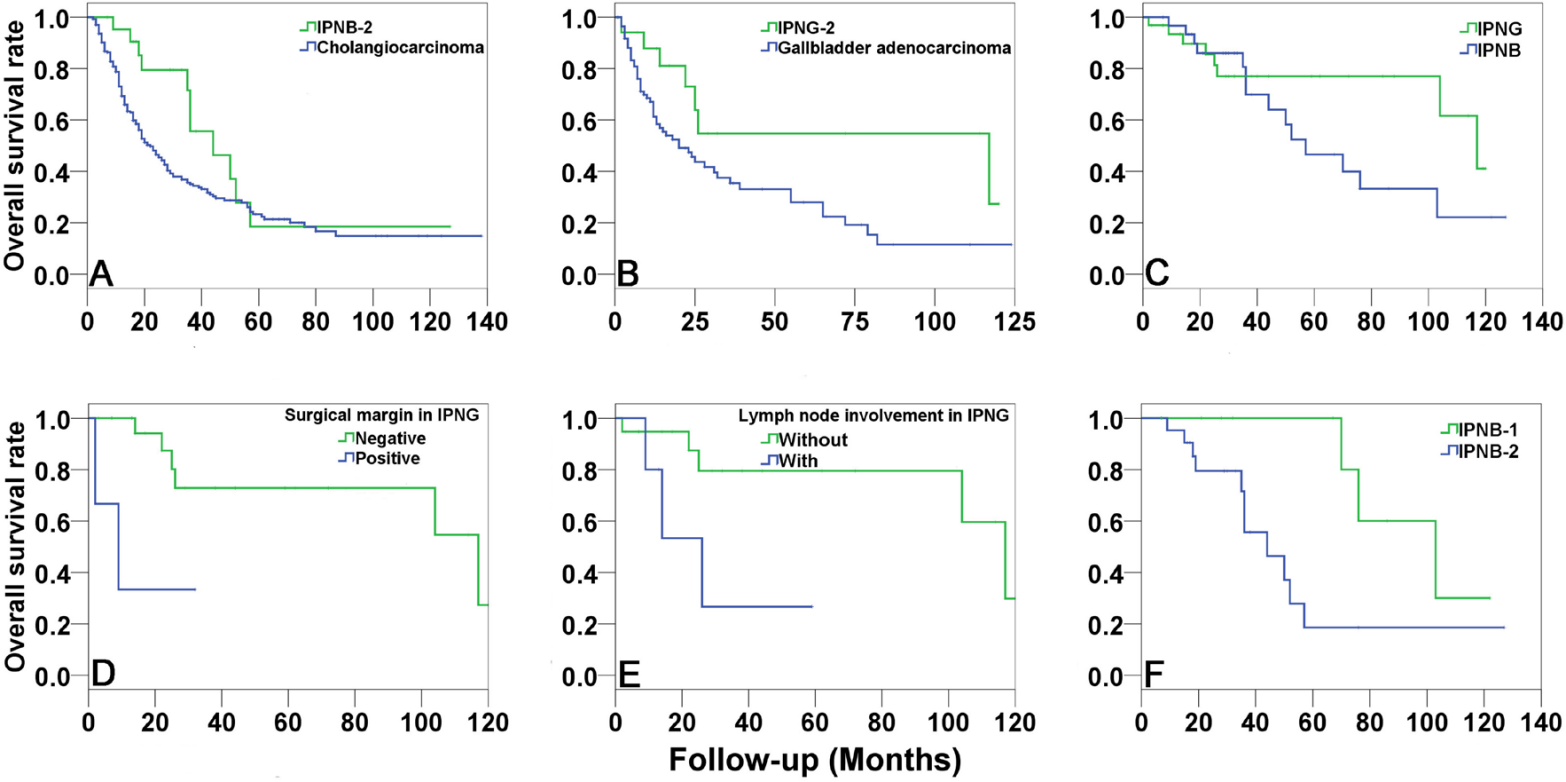
Survival analysis of IPNG and IPNB The overall survival of IPNB-2 (**A**) and IPNG-2 (**B**) were significantly better than that of cholangiocarcinoma and gallbladder adenocarcinoma, respectively. There was no significant difference in overall survival between IPNB and IPNG (**C**). Multivariate analysis suggested that positive surgical margin (**D**) and lymph node involvement (**E**) were independent risk factors of IPNG. However, the independent risk factor of IPNB was invasive carcinoma (**F**).

The median overall survival of IPNB-2 was 44.0 months (95% CI, 23.5–64.6 months), with 1-, 3-, and 5-year survival rates of 95.2%, 55.6%, and 18.5%, respectively, which was better than that of cholangiocarcinoma, whose median overall survival was 22.0 months (95% CI, 17.6–26.4 months), with 1-, 3-, and 5-year survival rates of 69.3%, 35.0%, and 23.3%, respectively (*P* = 0.047, Figure 5A). Similarly, the median overall survival of IPNG-2 was 117.0 months (95% CI, 15.1–218.9 months), with 1-, 3-, and 5-year survival rates of 87.8%, 54.7%, and 54.7%, respectively. However, the median overall survival of gallbladder adenocarcinoma was only 20.0 months (95% CI, 10.2–29.8 months), with 1-, 3-, and 5-year survival rates of 67.1%, 35.4%, and 28.0%, respectively (*P* = 0.033, Figure 5B). The median overall survival of IPNG as a whole was 117.0 months (95% CI, 92.6–141.4 months), with 5- and 10-year survival rates of 77.0% and 41.1%, respectively. The median overall survival of IPNB was 57.0 months (95% CI, 32.2–81.8 months), with 5- and 10-year survival rates of 46.6% and 22.2%, respectively. However, the difference was not significant (*P* = 0.172, Figure 5C).

Multivariate analysis suggested that positive surgical margin and lymph node involvement were independent risk factors of IPNG. The median overall survival of patients with negative surgical margin was 117.0 months, which was much longer than that of positive cases (9.0 months, *P* = 0.023, Figure 5D), with a hazard ratio of 8.7 (95% CI, 1.3–56.7). The median overall survival of cases without lymph node involvement was also much longer than that of cases with lymph node involvement (117.0 vs. 26.0 months, *P* = 0.021, Figure 5E), with a hazard ratio of 7.6 (95% CI, 1.4–42.1). However, the independent risk factor of IPNB was invasive carcinoma (*P* = 0.040, Figure 5F). The median overall survival of IPNB-1 was 103.0 months (95% CI, 60.9–145.1 months), which was much longer than that of IPNB-2 (44.0 months), with a hazard ratio of 2.0 (95% CI, 1.0–3.8).

## DISCUSSION

IPNB is widely recognized as a pathological entity that includes papillary cholangiocarcinoma and precursor lesions. Our findings demonstrated that IPNB with or without invasive carcinoma exhibited nearly identical clinical features, and gradual changes in histological subtypes and immunohistochemical expression files during tumor progression. For example, the main histological subtype of IPNB-1 was pancreaticobiliary (77.8%), and most IPNB-2 cases were classified as intestinal subtype (62.5%). Furthermore, CDX2, CK20 and MUC2, which are known as markers of intestinal metaplasia [4, 10–12], were expressed more frequently in IPNB-2 than in IPNB-1.

It is believed that adenomas of the gallbladder play a minor role in the pathway of gallbladder carcinogenesis, but some studies suggest that papillary adenoma is involved in the “adenoma-adenocarcinoma” process [13–15]. Gallbladder adenocarcinoma originating from adenoma is relatively uncommon may partly due to papillary adenoma accounts only a small portion of gallbladder adenoma [16, 17]. Several previous studies demonstrated that gallbladder papillary neoplasms appeared macroscopically as cauliflower-like masses projecting into the lumen of the gallbladder and microscopically as papillary proliferation of epithelial cells with delicate fibrovascular stalks, and the prognosis of gallbladder papillary adenocarcinoma was better than that of gallbladder adenocarcinoma (NOS), which was extremely similar to the presentation of IPNB [18–20].

Therefore, we performed comparative analyses between IPNG-1 and IPNG-2 to investigate the relationship between gallbladder papillary adenoma and adenocarcinoma. Clinicopathologically, the mean tumor size of IPNG-2 was 4.0 ± 2.2 cm, which was much larger than that of IPNG-1 (1.5 ± 1.2 cm, *P* < 0.001). The average onset age of IPNG-1 was 56.5 ± 16.9 years, which was younger than that of IPNG-2. These findings suggested that IPNG-1 was an early stage of IPNG. The IPNG-1 lesions grew gradually over time, gained invasiveness, and progressed to IPNG-2. Moreover, the distribution of histological subtype was similar in IPNG-1 and IPNG-2 and in all of the immunohistochemical markers except CK20, which was expressed more frequently in IPNG-2 than in IPNG-1. These findings were consistent with previous studies focused on IPNB, which demonstrated that CK20 was expressed more frequently in intraductal papillary neoplasm of the liver than non-neoplastic bile ducts and non-papillary intrahepatic cholangiocellular carcinoma, and its incidence increased significantly in parallel with lesion progression [21, 22]. The younger onset age and smaller tumor size of IPNG-1 and the increased expression of CK20 during tumor progression suggest that gallbladder papillary adenoma and adenocarcinoma, which were separated in clinical practice, can be different stages of a single pathological entity, which we propose to name intracystic papillary neoplasm of the gallbladder.

Thereafter, we compared IPNG to IPNB and found them with great similarities. Firstly, pancreaticobiliary and intestinal subtype accounted for nearly half of IPNG or IPNB (44.4% and 48.1% vs. 44.0% and 44.0%), respectively. Secondly, the distribution of immunohistochemical expression of MUCs, CKs, and CDX2 were similar, and MUC1 and CK7 expression were prevalent. Thirdly, CDX2 and MUC2 were more highly expressed in the intestinal subtype than in other subtypes. Fourthly, CDX2 expression was positively correlated with MUC2 and CK20 expression. Fifthly, 53.1% of IPNG cases and 68.6% of IPNB cases exhibited invasive carcinoma, and showed similar significant survival advantages to conventional gallbladder adenocarcinoma and cholangiocarcinoma, respectively. Sixthly, CK20 expression increased in parallel with tumor progression.

On one hand, 88.9% of IPNG and 92.0% of IPNB cases were positive for MUC1, and 96.3% and 92.0% for CK7, respectively. MUC1, a marker of pancreaticobiliary differentiation and reported to be associated with the pancreaticobiliary subtype of IPNB in previous studies [1, 3], was prevalent in almost all cases in our study. CK7, as a marker of biliary epithelium [23], was also expressed in almost every case of IPNG and IPNB. Therefore, we speculated that IPNG and IPNB likely retained pancreaticobiliary phenotype (MUC1/CK7) during tumorigenesis. On the other hand, CDX2 and MUC2 expression levels in IPNG or IPNB were associated with the intestinal subtype, and CDX2 was positively correlated with MUC2 and CK20 expression, which is consistent with previous studies [1, 2, 4, 11, 12, 22] and suggests that intestinal metaplasia plays a role in the tumorigenesis of IPNG and IPNB. The immunohistochemical markers CDX2 and CK20 in IPNB and CK20 in IPNG were associated with invasive carcinoma, which suggests that intestinal metaplasia participated in tumorigenesis, and some internal molecular changes of this pathological process also play a role in tumor progression. The specific intrinsic mechanisms may be heterogeneous in tumors originating from different locations.

## MATERIALS AND METHODS

### Case selection and clinicopathological data

Surgical pathology files from January 2003 to August 2014 of the Peking Union Medical College Hospital were retrieved. A total of 32 cases of gallbladder tumors that were papillary or contained papillary ingredients (10 adenoma and 22 adenocarcinoma in accordance with previous diagnostic criteria) and 32 cases of IPNB (7 intrahepatic, 8 hilar, and 17 extrahepatic cases) were included. We also included 97 cases of gallbladder adenocarcinoma (NOS) and 303 cases of cholangiocarcinoma (NOS, 92 intrahepatic, 79 hilar, and 132 common bile duct cases) from the same period for comparative survival analyses. Diagnoses were re-confirmed, and 15 of 32 IPNG cases were identified as low-high-grade dysplasia (IPNG-1). The other 17 cases were diagnosed with invasive carcinoma (IPNG-2). Ten of the 32 IPNB cases were low-high-grade dysplasia (IPNB-1), and the other 22 cases were invasive carcinoma (IPNB-2). Clinicopathological data of all patients were obtained from medical records. All participants provided written informed consent, and the Peking Union Medical College Hospital Ethics Committee approved all study procedures.

### Immunohistochemical staining

Formalin-fixed and paraffin-embedded tissue blocks were available in 27 cases of IPNG (12 IPNG-1 and 15 IPNG-2) and 25 cases of IPNB (9 IPNB-1 and 16 IPNB-2), and one or two representative blocks from each case were subjected to immunohistochemical staining. Expression of MUC1, MUC2, CK7, CK20 and CDX2 was classified semiquantitatively into four scores according to the percentage of positive cells in the individual lesion: negative (-), 0%; focal (+), 1% to 10%; moderate (++), 11–50%; and diffuse (+++), more than 50%.

### Statistical analysis

Comparisons of continuous variables were performed using Student's *t* test. Comparisons of categorical variables were performed using the Pearson chi-square test and Fisher's exact test. Spearman's rank correlation test was used to evaluate the correlation between categorical variables. Kaplan-Meier curves and the log-rank tests were used to compare overall survival. Cox regression analysis was performed to identify prognostic factors in each group. Statistical significance was set at *P* < 0.05.

## CONCLUSIONS

Papillary adenoma and adenocarcinoma of the gallbladder can be recognized as tumors at different stages of a single pathological entity, namely, IPNG. The distribution of histological subtypes and immunohistochemical phenotype of IPNG resembled that of IPNB. During tumorigenesis, IPNG and IPNB likely retained pancreaticobiliary phenotype (MUC1/CK7), and nearly half of the cases obtained intestinal phenotype (CDX2/MUC2/CK20), and intrinsic molecular changes in the process of intestinal metaplasia such as CK20 expression may contribute to the progression of these tumors.

## Abbreviations

IPNB: intraductal papillary neoplasm of the bile duct
IPNG: intracystic papillary neoplasm of the gallbladder.
